# Upstream Open Reading Frames Located in the Leader of Protein Kinase Mζ mRNA Regulate Its Translation

**DOI:** 10.3389/fnmol.2016.00103

**Published:** 2016-10-13

**Authors:** Natalia V. Bal, Denis Susorov, Ekaterina Chesnokova, Artem Kasianov, Tatiana Mikhailova, Elena Alkalaeva, Pavel M. Balaban, Peter Kolosov

**Affiliations:** ^1^Cellular Neurobiology of Learning Laboratory, Institute of Higher Nervous Activity and Neurophysiology, Russian Academy of SciencesMoscow, Russia; ^2^Laboratory of Mechanisms and Control of Translation, Engelhardt Institute of Molecular Biology, Russian Academy of SciencesMoscow, Russia; ^3^Faculty of Bioengineering and Bioinformatics, M. V. Lomonosov Moscow State UniversityMoscow, Russia; ^4^Laboratory of System Biology and Computational Genetics, Vavilov Institute of General Genetics, Russian Academy of SciencesMoscow, Russia

**Keywords:** PKMζ, uORF, local protein synthesis, translational control, eIF2a phosphorylation

## Abstract

For protein synthesis that occurs locally in dendrites, the translational control mechanisms are much more important for neuronal functioning than the transcription levels. Here, we show that uORFs (upstream open reading frames) in the 5′ untranslated region (5′UTR) play a critical role in regulation of the translation of protein kinase Mζ (PKMζ). Elimination of these uORFs activates translation of the reporter protein *in vitro* and in primary cultures of rat hippocampal neurons. Using cell-free translation systems, we demonstrate that translational initiation complexes are formed only on uORFs. Further, we address the mechanism of translational repression of PKMζ translation, by uORFs. We observed an increase in translation of the reporter protein under the control of PKMζ leader in neuronal culture during non-specific activation by picrotoxin. We also show that such a mechanism is similar to the mechanism seen in cell stress, as application of sodium arsenite to neuron cultures induced translation of mRNA carrying PKMζ 5′UTR similarly to picrotoxin activation. Therefore, we suppose that phosphorylation of eIF2a, like in cell stress, is a main regulator of PKMζ translation. Altogether, our findings considerably extend our understanding of the role of uORF in regulation of PKMζ translation in activated neurons, important at early stages of LTP.

## Introduction

It is known that two-thirds of genes expressed in humans are necessary for neural tissue functioning (Uhlén et al., 2015). It is also a well-established fact that neuronal cells are very elongated and highly polarized, with different proteins accumulating in axons, dendrites, and soma. Moreover, at any given moment the protein content of a neuron may be different depending on its activation status, signals from other neurons, extracellular conditions, and many other factors. Taken together, these facts suggest that precise spatial- and temporal-specific regulation of protein synthesis is necessary for normal neuronal functioning.

It has been demonstrated that blocking mechanisms of translation are much more important for neuronal functioning than transcription arrest, because protein translation happens locally, in the proximal parts of dendrites (Ho et al., 2011). Downregulation of translation may occur by different means: mRNA-binding proteins like FMRP (Panja et al., 2014), small non-coding RNAs (BC1; Eom et al., 2014), stalled translation initiation complexes, (Graber et al., 2013) and phosphorylation of different translation factors (Bellato and Hajj, 2016). Despite the intensive research in this field, the fine molecular mechanisms of synaptic protein translation regulation are not fully determined yet.

Recently published data (Sacktor, 2012; Tsokas et al., 2016) suggest the involvement of such regulation in the functioning of the atypical kinase protein kinase Mζ (PKMζ). PKMζ has been shown to be necessary for LTP formation (Ling et al., 2002; Pastalkova et al., 2006; Tsokas et al., 2016). It was demonstrated (Kelly et al., 2007) that PKMζ participates in many signaling pathways that are involved in LTP (PI3-kinase, CaMKII, MAPK, PKA, mTOR cascades), and that the PKMζ concentration in the postsynapse must increase for successful LTP formation. PKMζ is one of the key regulators of synaptic plasticity and memory formation. Neuronal stimulation causes an increase in translation of its mRNA in postsynapses (Sacktor, 2012). The peculiarity of the PKMζ molecule is that its selective blockade causes impairment of long-term memory but doesn’t affect the formation of new memories (Shema et al., 2007; Serrano et al., 2008; von Kraus et al., 2010). Recently (Tsokas et al., 2016) it was demonstrated that while PKMζ maintains late-LTP and long-term memory in wild-type mice, the PKCι/λ, a gene-product closely related to PKMζ, persistently increases in LTP maintenance in PKMζ-null mice. This eliminates a controversy with data indicating that PKMζ may be not crucial for memory maintenance (Lee et al., 2013; Volk et al., 2013). Additional evidence of PKMζ necessity for memory storage in invertebrates has been published recently (Balaban et al., 2015) and its role in memory has been shown without inhibitors, using the effect of PKMζ overexpression in the hippocampus on behavior and LTP in rats (Schuette et al., 2016).

PKMζ is an unusual member of PKC kinases family because it does not have a regulatory domain. The lack of regulatory subunit within the PKMζ molecule is the reason for its constitutive kinase activity. PKMζ activity levels can be regulated by changes in its translation efficiency and, to a lesser degree, by its phosphorylation. It was supposed that if a neuron is inactive, the level of PKMζ is controlled by translation inhibition mechanisms (Sacktor, 2012). However, it’s still not clear how exactly this suppression is realized.

It is thought that translation is more crucial to the increase of amount of PKMζ than transcription, because the level of *de novo* synthesized PKMζ grows within a few minutes of tetanization, but the concentration of PKMζ mRNA doesn’t change even after an hour (Kelly et al., 2007). A positive feedback model has been suggested, in which PKMζ may facilitate its own translation. PDK1 kinase is necessary for this process. PDK1 phosphorylates PKMζ at T410, and, in addition, PKMζ phosphorylates itself at T560. Phosphorylation increases the kinase activity of PKMζ. Moreover, phosphorylation of the PKMζ targets indirectly facilitates the translation of several proteins, PKMζ itself included, via different signaling pathways (Westmark et al., 2010).

Several mechanisms, by which PKMζ translation may be downregulated normally and upregulated due to synaptic stimulation, have been proposed. According to one of the suggestions, translational inhibition could be linked with the peptidil-prolil isomerase Pin1 activity. If Pin1 is knocked out, the PKMζ level increases (Westmark et al., 2010). There are also data confirming that the PKMζ translation may be regulated by BC1 RNA and cis-regulated by highly the structured 5′ untranslated region (5′UTR) of PKMζ mRNA (Hernandez et al., 2003; Eom et al., 2014). For example, it was shown that truncation of the leader sequence of PKMζ increases translation of the main open reading frame (Hernandez et al., 2003).

It is known that not only the length and secondary structure, but also some other properties of 5′UTR may affect the translation of mRNA. One such factor is the presence of upstream open reading frames (uORFs) in the leader sequence. According to the scanning model, a 43S preinitiation complex, which consists of the 40S ribosome subunit, Met-tRNA-eIF2-GTP, and initiation factors eIF3, eIF1, eIF1A, eIF5, recruits to mRNA by binding to m^7^G cap via initiation factor eIF4F (eIF4E, eIF4G, and eIF4A). This complex scans 5′UTR and recognizes the start codon [for a recent review (Hinnebusch, 2014)]. Translation of the next ORFs is complicated because it requires re-initiation or another mechanism. Thus uORFs are able to competitively inhibit the translation of the main ORF by binding the translation apparatus components in 5′UTR. It is also known that the inhibiting effect of uORFs may be attenuated under specific conditions including phosphorylation of the α subunit of translation initiation factor eIF2 (eIF2α). Factor eIF2 is critical for start codon selection. In the ternary complex with GTP and MetRNA, eIF2 recognizes the start codon. After GTP hydrolysis, eIF2^∗^GDP releases from the ribosome. Another round of initiation begins when eIF2-GDP is converted to eIF2-GTP by eIF2B. Phosphorylation of eIF2α prevents rapid recycling of GTP by eIF2B (O’Connor et al., 2008). There are four different kinases able to phosphorylate eIF2α: GCN2, PKR, PERK, and HRI. All of them are involved in IGR (Integrated General Stress). Recently RiboSeq was performed on the cells treated by sodium arsenite (Andreev et al., 2015). The translation of most mRNAs was significantly suppressed by treatment with sodium arsenite, which triggered phosphorylation of eIF2α. Only a small set of mRNA was not suppressed and all of them contained uORFs. Under stress conditions, the translation of uORFs decreased whereas the translation of the main ORFs was modestly enhanced or at least did not change (Andreev et al., 2015).

Considering learning and synaptic plasticity, in experiments *in vivo* it has been shown that the knockout of eIF2α phosphorylating kinase genes or chemical inhibition of these kinases resulted in enhanced memory and enhanced late LTP induction after weak stimulation. KO GCN2-/- mice after weak training accomplished a spatial task better than the wild-type animals. Strong training led to disruption/impairment of the spatial memory in KO GCN2-/- mice. In hippocampal slices obtained from KO GCN2-/- animals, translation-dependent late LTP (L-LTP) reached a significance level after a single 100-Hz train, while in the case of wild-type slices this protocol induced only early LTP (Costa-Mattioli et al., 2005; Zhu et al., 2011; Trinh et al., 2014). One can conclude that if eIF2α phosphorylation is blocked, the L-LTP-associated protein synthesis in neurons occurs after a single stimulation, and these proteins may cause long-term behavioral modifications. It is well known that eIF2α is able to switch the main translation capacity from uORF-free to uORF-containing mRNAs (Dever et al., 1992). We assume that, in KO GCN2-/- mice, synthesis of proteins, required for regulation of L-LTP/LTD, is controlled by uORFs. Due to the lack of phosphorylation of eIF2 in hippocampal neurons, the total protein synthesis is not suppressed in these mice, and LTP prevails over LTD. In wild-type mice, both activation of translation of mRNA, containing uORFs, and decrease of total protein synthesis, allow suppression of the redundancy of response to neuron stimulation. There are published data concerning uORFs participation in translation regulation of some proteins that are important for synaptic plasticity, like Shank1, SAPAP, or oligophrenin-1 (Chua et al., 2012; Di Prisco et al., 2014; Studtmann et al., 2014). uORF-dependence of PKMζ translation has not been studied in detail yet, while it could be critical for the regulation of synaptic plasticity and memory.

**FIGURE 1:**
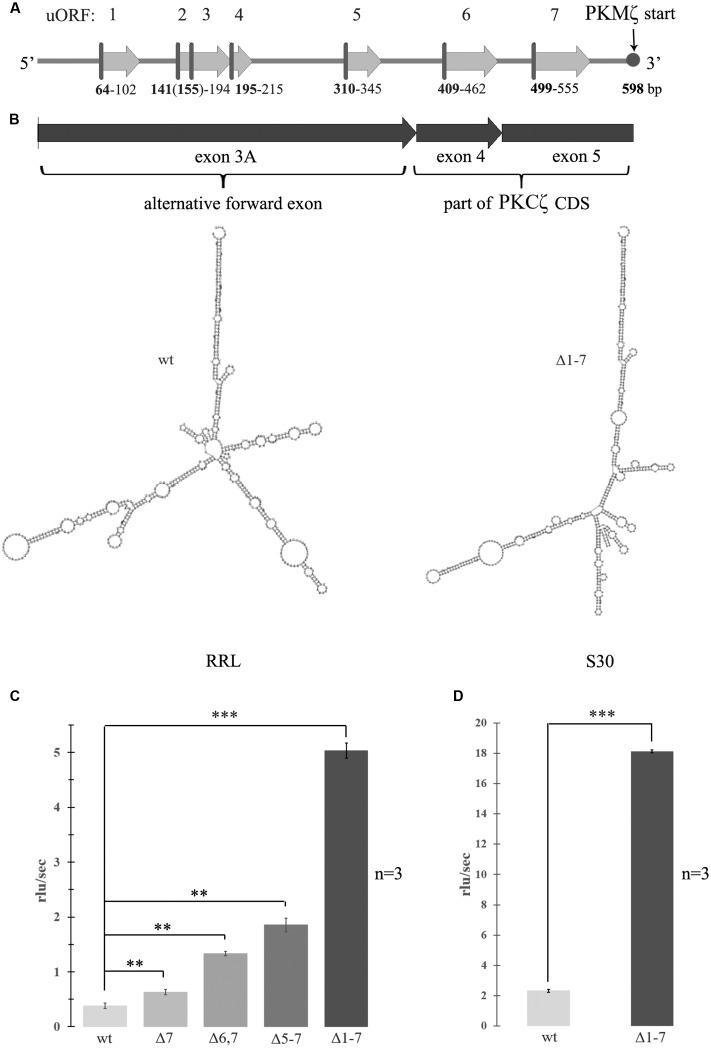
**Activation of translation by mutagenesis of upstream open reading frames (uORFs) in 5′ untranslated region (5′UTR) of the protein kinase Mζ (PKMζ).** **(A)** Structure of 5′UTR of mRNA of PKMζ. uORFs are indicated by numbers. **(B)** Secondary structure of wild-type or mutated leaders of PKMζ (in m-fold). left – wild-type; right – Δ1–7. Structures are have the same form with similar energies -227 ccal/mole. **(C)** Rates of translation of mRNAs containing different mutated leaders of PKMζ and CDS of luciferase in rabbit reticulocyte lysate (RRL). **(D)** Rates of translation of mRNAs containing different mutated leaders of PKMζ and CDS of luciferase in Crebs lysate (S30). mRNAs contain six (Δ7), five (Δ6,7), four (Δ5–7), and no (Δ1–7) uORFs. The error bars represent the standard deviation, asterisks indicate a significant difference determined by a *t*-test, ^∗∗∗^*P* < 0.001, ^∗∗^*P* < 0.01 (*n* = 3). Rlu, related luminescence units.

The mRNA encoding PKMζ has an extended 5′UTR (Hernandez et al., 2003). During the splicing process, the 5′UTR of PKMζ mRNA is modified, and its sequence is unique. Within this 5′UTR there are seven AUG codons, the potential starting points for uORFs (**Figure 1A**). In the present study, we have compared the effects of uORFs and the structure of mRNA on translation of PKMζ in different translation systems. We demonstrated that the formation of initiation ribosomal complexes on uORFs is the main factor for PKMζ translation regulation. If uORFs in the PKMζ 5′UTR were mutated, basic translation level of the reporter protein increased considerably in comparison to the wild-type 5′UTR sequence. In experiments with cell cultures, we showed that 5′UTR of PKMζ mRNA downregulated translation of the reporter protein, but after neuronal activation caused by picrotoxin, its translation was significantly increased. We propose that the molecular mechanism of the translational control of PKMζ mRNA is bound to regulation by uORF and phosphorylation of eIF2a.

## Materials and Methods

### Plasmid Constructs and *In vitro* Transcription

For experiments in living cells (in primary culture of hippocampal neurons), constructs based on the p156 vector were obtained. The non-integrated lentiviral vector p156RRL (rabbit reticulocyte lysate)–sin18-PPT-PRE (Terskikh et al., 2005) was kindly provided by Alon Chen (München, Germany). These constructs contained the PKMζ 5′UTR (wild-type or mutated) fused to mCherry followed by CRPV IRES and GFP as an internal control.

The constructs for mRNAs for *in vitro* experiments were prepared by insertion of PKMζ or actin 5′UTRs followed by the firefly luciferase coding sequence into pGEM-T vectors. PCR based site-directed mutagenesis was used to mutate AUG codons in the PKMζ 5′UTR.

PCR templates for synthesis of mRNAs for *in vitro* experiments were obtained directly from the pGEM-based constructs with the corresponding T7 promoter-containing forward primers and sequence specific reverse primers (Dmitriev et al., 2007). The 50 μl transcription mixture contained 40 mM Hepes-KOH, 1 mM spermidine, 25 mM MgCl2, 1 mM DTT, 5 mM 4NTPs, 1 u/mkl Ribolock, 5 u/μl T7 RNA polymerase, and 200 ng/μl of the PCR template. The resulting transcripts were precipitated with 2 M LiCl. For all transcripts, the Vaccinia Capping System (NEB) was used to obtain 100% capped products.

### Computational Analysis of uORF Distribution

Rnor 6.0 genome version of *Rattus norvegicus* assembly and GRCm38 genome version of *Mus musculus* were used. For rat genes, the 5′ UTR regions were defined as the longest regions from annotated mRNA start to the appropriate annotated CDS start. For mouse genes, annotated 5′ UTR regions were extracted. ORF in UTR regions were found by using EMBOSS getorf tool. The number of UORF for each gene or specified group of genes was counted using a homemade script. The results of computational analysis are summarized in **Supplementary Figure S1**.

### *In vitro* Translation

*In vitro* translation was performed in Krebs-2 cells S30 extract or in RRL (Promega), in total volumes of 10 and 20 μl, respectively (Terenin et al., 2016). Reaction mixtures contained 5 μl of the S30 extract or 10 μl of the RRL, translation buffer (20 mM Hepes-KOH pH 7.6, 1 mM DTT, 0.5 mM spermidine-HCl, various amounts of Mg(OAc)_2_ (1–3.5 mM), 8 mM creatine phosphate, 1 mM ATP, 0.2 mM GTP, various amounts of KOAc (40–120 mM) and 25 μM of each amino acid), 2 u of RiboLock RNase inhibitor (Thermo Scientific), 0,1 mM luciferin, 300 ng of the PKMζ 5′UTR mRNA or 75 ng of the actin 5′UTR Mrna, and in some cases 5 pmol of initiation factors (eIF1, eIF1A, eIF2, eIF4A, eIF4B, eIF4G, or ΔeIF4G). The kinetics of luciferase synthesis was measured using a Tecan Infinite200pro at 30°C for 30 min.

### Toe-Print of Ribosomal Complexes in RRL

To assemble ribosomal complexes, we utilized commercially available RRL (Promega). We followed the previously published protocol (Dmitriev et al., 2003) with some modifications. The reaction was initiated in a total volume of 9 μl containing 7 μl RRL, 1 mM Mg2+, 0.5 u/μl Ribolock and 75–200 ng of mRNA, and was incubated for 5 min at 30°C. After that, 10 μl of RT Mix were added directly into the tube at 30°C. The RT Mix contained 20 mM Tris-HCl pH 7.5, 60 mM KCl, 0.25 mM spermidine-HCl, 1 mM DTT, 2 μl of dNTP mix (5 mM each), 1 μl of 6-carboxyfluorescein-labeled oligonucleotide (5 pmol/μl, 5′-FAM-GATGTTCACCTCGATATG-3′), 1 μl of 0.5 M Mg(OAc)_2_, 1 μl of AMV Reverse Transcriptase (Promega), and 4 μl of water. The mixture was incubated for 15 min at 30°C. The resulting cDNAs were then purified by thorough phenol/chloroform extraction, precipitated with ethanol, dissolved in 100%-formamide and analyzed by capillary gel electrophoresis in an ABI PRISM 3100-Avant Genetic Analyzer (Applera).

### Preparation of Hippocampal Primary Neuron Culture and Subsequent Transfection

For each test, three experimental and three control cultures were used. All experimental procedures were conducted in accordance with the European Communities Council Directive of 24 November 1986 (86/609/ EEC) on the protection of animals used for scientific purposes. The study protocol was approved by the Ethics Committee of the Institute of Higher Nervous Activity and Neurophysiology of RAS. Wistar rat pups (P0–P2) were euthanized by decapitation with sharp scissors. The brains were removed, hippocampi dissected and gently cut into pieces with a sharp blade in ice-cold DMEM solution (Paneco) with glutamine. Next, the tissue was treated in DMEM with trypsin (10 mg in 12,5 ml of solution) for 15 min at 36°C and then centrifugated at 2000 rpm for 2 min. Cells were washed with the Neurobasal medium followed by centrifugation at 2000 rpm for 2 min two times. Then the tissue was resuspended in Neurobasal Medium (Gibco) with 2% B-27 supplement (Gibco), GlutaMax (Gibco), and cells were placed onto 12 mm glass coverslips coated with poly-D-lysine (SIGMA). Cultures were housed in a CO_2_ incubator at 5% CO_2_ concentration and 37°C temperature prior to the fluorescent imaging experiments.

At the seven DIV, neurons were transfected by plasmids using Lipofectamine 2000 (Invitrogen). The following day, picrotoxin (to a final concentration of 30 μM) or a control solution was added to culture. Fluorescent images were obtained at nine DIV using microscope Keyence BZ-9000 (Japan) and analyzed with ImageJ (NIH). For quantitative comparison of mCherry expression in different neurons, the fluorescence of the proximal parts of dendrites was measured. The length of measured region was 35 μm. This part was chosen for standardization of quantitative analysis. The measurement area was identical and it was located at an equal distance from the soma in all cells. The red fluorescence/green fluorescence ratio was calculated after background subtraction.

### Polyacrylamide Gel Electrophoresis and Western Blotting

Cell cultures were prepared from neonatal rat hippocampi as described above. At the 14 DIV picrotoxin (to a final concentration of 30 μM) or control solution was added to culture. After 12 h, cells were harvested and resuspended in 1000 μl of cold (4°C) phosphate buffer saline (PBS). Next, cell suspensions were centrifuged at 1000 *g*, 3 min, at 4°C. The pellet was resuspended in 25 μl of cold PBS and then mixed with 25 μl of 2^x^ Laemmli sample buffer. Each sample was divided into two halves, in one half eIF2α and in the other p-eIF2α concentrations were detected.

Samples were incubated at 95°C for 10 min, loaded to 12% TGX Stain-Free FastCast acrylamide gel (Bio-Rad) and electrophoresed according to the supplier recommendations. Total protein levels were estimated in the gels using the ChemiDoc Touch Imaging System (Bio-Rad), Stain-Free assay protocol. Proteins were then transferred from gels to PVDF membranes by electrophoresis with the standard program for TGX gel for 3 min. The SNAP i.d.^®^ 2.0 Protein Detection System (Millipore) was used to shorten the time required for blocking, washing, and antibody incubations. After blocking with 0.5 % no fat milk (in PBS+0.1% tween-20, 30 ml) for 10 min, membranes were incubated in 5 ml of primary antibodies solution [rabbit antibodies to eIF2α (Cell Signaling, #5324) or p-eIF2α (Cell Signaling, #3398) diluted 1:1000 in milk] for 10 min, then washed with 120 ml of PBS+tween for 5 min. For incubation with secondary antibodies [goat anti-rabbit conjugated with horseradish peroxidase (Bio-Rad, #172–1019), diluted 1:1000 in milk] and their washing the same protocol as for primary antibodies was used.

Bands were detected with the ECL Advance Western blotting detection kit (GE Healthcare) on the ChemiDoc Touch Imaging System, chemiluminescence assay. Immunoblot signals were quantified by densitometric scanning and image analysis with Image Lab Software (Bio-Rad). Optical density of eIF2α and p-eIF2α bands was normalized to total protein in the lane. After normalization, the p-eIF2α/eIF2α ratio was calculated for each sample. Samples obtained from four independent experiments.

## Results

### uORFs in 5′UTR of PKMζ Inhibit Cell-Free Translation

To examine the effect of the structure and composition of 5′UTR of PKMζ mRNA on its translation, we designed genetic constructions containing substitutions of start codon ATG to stop codon TAG of each uORF in 5′UTR. Thus, we “turned off” all seven uORFs of 5′UTR PKMζ in consecutive order (**Figure 1A**). As a result, we obtained seven genetic constructions that had the same length but contained different numbers of uORFs. The modeled secondary structures of “wild-type” 5′UTR and Δ1–7 construct, with disabled uORFs, have the same forms with similar energies -227 ccal/mole (**Figure 1B**). This means that “turning off” the uORFs does not significantly change the overall structure of mRNA. To estimate the efficiency of translation of these constructions *in vitro* (in cell lysates), the mutated 5′UTRs were inserted into pGEM-T plasmid encoding luciferase. The efficiency of translation of the obtained mRNAs was detected in RRL (**Figure 1C**) and mouse ascite carcinoma cell lysate (S30; **Figure 1D**).

We found that translation of the main ORF was more effective if 5′UTR contained a reduced numbers of uORFs. This result corresponds to the concept of translation inhibition by uORFs. It was shown previously (Hernandez et al., 2003) that shortening the 5′UTR of PKMζ increased luciferase translation *in vitro*. This may be caused by reduction in the number of uORFs or by changes in mRNA secondary structure (unfolding; Hernandez et al., 2003; Barbosa et al., 2013). Our data demonstrate that the translation rate depends on the presence of uORFs that compete for translation initiation complexes with the main coding frame, and is not affected by the secondary structure of the leader sequence.

We demonstrated the importance of uORFs for the translational control of PKMζ. The question then arises whether this mechanism of regulation is a basic one for neurons. To answer the question we performed bioinformatics analysis of the available soma and dendritic associated transcriptome data sets for mouse (Ainsley et al., 2014). In this research, only neuronal mRNAs from hippocampal CA1 region were investigated. We conducted a comparison of neuronal and non-neuronal whole transcriptome data sets (**Supplementary Figure S1A**). The neuronal data set combines soma and dendritic associated transcriptomes. In this case, we observed a difference in the percentage of expressed genes, which was dependent on the number of uORFs in mRNA. The percentage of expressed genes with 0–2 uORFs is smaller in the neuronal transcriptome than in the whole transcriptome. On the contrary, the amount of expressed genes with 3–10 uORFs is greater in the neuronal fraction compared to the whole transcriptome (*p*-value < 0.0001, Chi square test). This result suggests that regulation of translation by uORFs is widely used in neurons.

Moreover, we carried out a comparison of the neuronal transcriptome and Ribo-seq data obtained 5 min after learning of *M. musculus* whole hippocampus (the sample contains neuronal and non-neuronal cells; Cho et al., 2015) (**Supplementary Figure S1B**). We determined that after learning the amount of translated mRNAs containing a large number of uORFs increases, and the amount of translated mRNAs without or with small number of uORFs decreases (*p*-value < 0.0287, Chi square test). This indicates a possible involvement of uORFs into translational control during memory consolidation.

### Conditions in Resting Neurons Are Not Optimal for Translation of PKMζ

**FIGURE 2:**
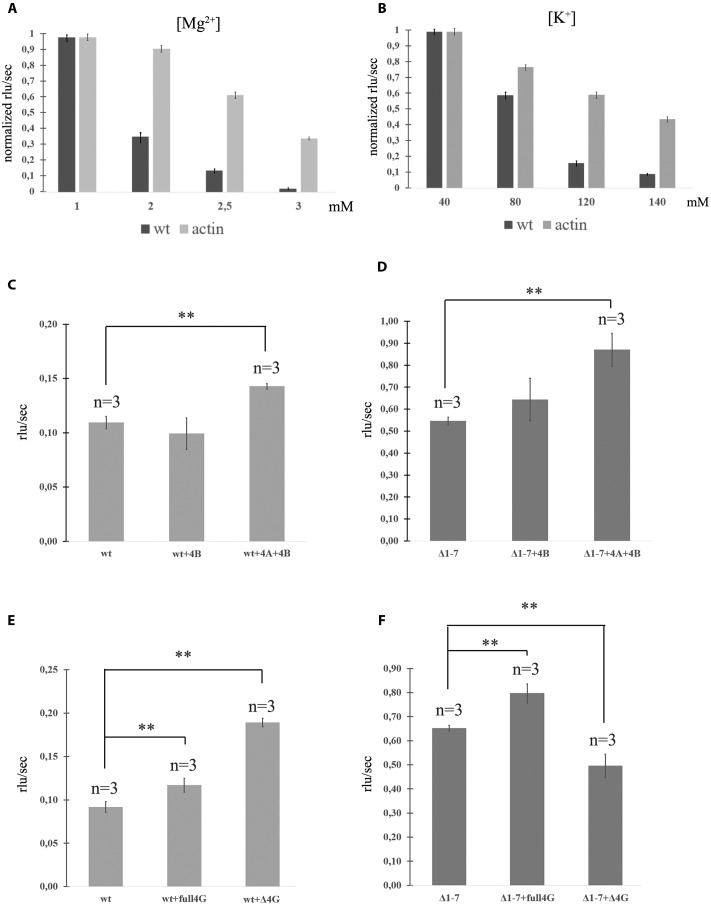
**Translation of mRNAs with wild-type and mutated PKMζ 5′UTR in RRL.** **(A)** Rates of translation of mRNAs containing PKMζ or actin 5′UTRs in different concentrations of Mg^2+^. **(B)** Rates of translation of mRNAs containing PKMζ or actin 5′UTRs in different concentrations of K^+^. **(C,D)** Rates of translation of mRNA containing wild-type **(C)** or mutated Δ1–7 **(D)** 5′UTR of PKMζ in the presence of eIF4B and eIF4A. **(E,F)** Rates of translation of mRNAs containing wild-type **(E)** or mutated Δ1–7 **(F)** 5′UTRs of PKMζ in the absence or presence of eIF4G and its truncated form Δ eIF4G. The error bars represent the standard deviation, asterisks indicate a significant difference determined by a *t*-test, ^∗∗^*P* < 0.01 (*n* = 3). Rlu, related luminescence units.

Another possibility for regulation of the translation efficacy may be a control of the level of ion concentrations in cells. As translation of PKMζ in non-activated neurons is suppressed, we decided to determine the contributions of the physiological concentrations of K^+^ and Mg^2+^ into such inhibition. We measured the efficiency of translation of model mRNA, which contains 5′UTR of PKMζ and the luciferase coding sequence, as a function of the concentration of cations (both K^+^ and Mg^2+^) in the cell lysate (**Figures 2A,B**). As a control, mRNA with 5′UTR of actin followed by luciferase CDS was used. We observed that the optimal cation concentrations for translation of mRNA with PKMζ 5′UTR were very narrow.

The efficiencies of translation of both mRNAs in 1 mM Mg^2+^ were identical and maximal (**Figure 2A**). However, increasing of the magnesium concentration to 3 mM lead to significant suppression of translation of mRNA with PKMζ 5′UTR. The translation of the control (actin) mRNA remained high. Interestingly, concentrations of Mg^2+^ in dendrites may vary (Cheng and Reynolds, 2000; Palacios-Prado et al., 2014). There may be local oscillations of Mg^2+^ concentration in neurons between 0.2 and 3.5 mM due to the reversible ATP-Mg^2+^ binding (Palacios-Prado et al., 2014). Therefore, different energy-consuming intracellular processes, including action potential generation or postsynapse activation, may cause an increase of the local Mg^2+^ concentration because of ATP consumption.

A similar effect was observed with various concentrations of K^+^ in the translation mixture (**Figure 2B**). If the K^+^ concentration in the solution was equal to the normal intracellular level (140 mM; Müller and Somjen, 2000), the translation efficacy was low. It constituted only 15% of the efficacy level that was observed if there was 80 mM K^+^ in the solution (**Figure 2B**). Translation of the control mRNA was slightly influenced by the potassium concentration. Consequently, the translation of PKMζ in normal concentrations of K^+^ and Mg^2+^ (140 and 2,5 mM, respectively) is suppressed and requires additional activation.

### uORFs Accumulate Translation Initiation Complexes on the 5′UTR of PKMζ

**FIGURE 3:**
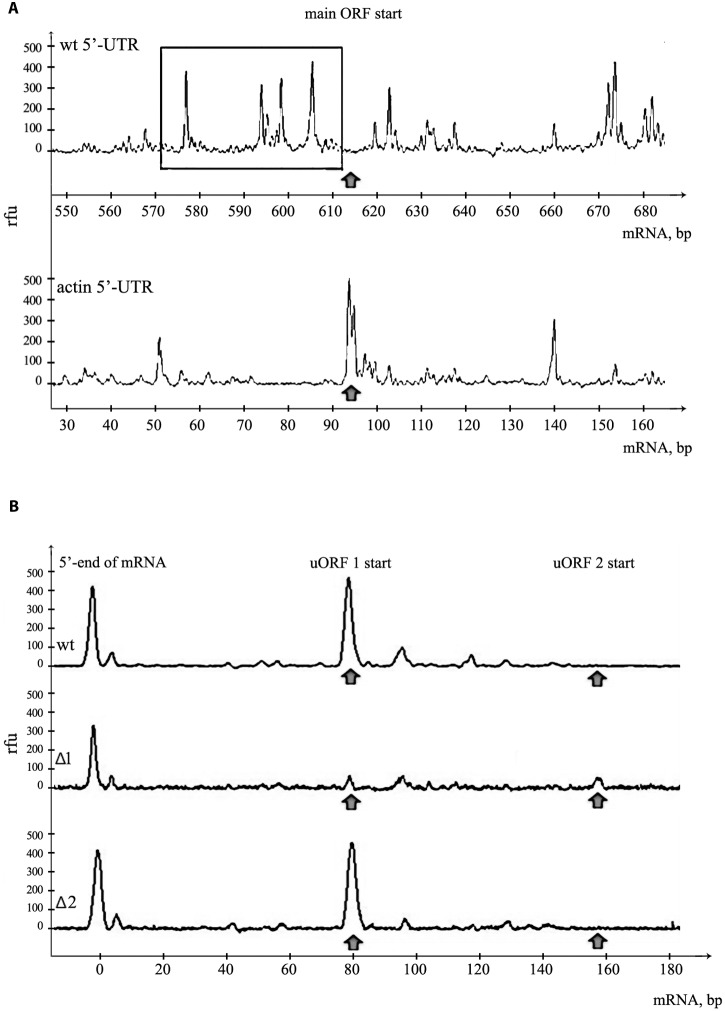
**Assembly of 48S initiation complexes on mRNA with PKMζ 5′UTR.** **(A)** Toe-print analysis of ribosomal complexes on mRNAs containing PKMζ or actin leader sequences and main ORF of luciferase. The area adjacent to the start of the main ORF is shown. Arrow indicates the position of AUG codon of the main ORF. Hairpins on 5′UTR of PKMζ are selected by rectangle. **(B)** Toe-print analysis of mRNAs containing wt and mutated leaders of PKMζ (Δ1 and Δ2). The beginning of 5′UTR is shown. Rfu, related fluorescence units. Arrows indicate the positions of 48S initiation complexes on uORFs.

The 5′UTR of PKMζ is 600 nt long and very structured. We have already shown that disabling uORFs does not affect its structure (**Figure 1B**). To confirm the high complexity of this sequence we applied the toe-printing analysis of mRNA with firefly luciferase coding sequence and 5′UTR of PKMζ in RRL (**Figure 3A**). The translation initiation complexes assembled on the mRNA in cell lysate could be detected by reverse transcription with the fluorescently labeled primer complimentary to the 3′-end of the RNA. The lengths of obtained cDNA fragments reflect the positions of ribosomal complexes or hairpins on the mRNA. We observed that the leader of PKMζ has many hairpins near the main ORF that prevent the movement of reverse transcriptase in the toe-printing assay. Furthermore, we didn’t detect any translation initiation complex on the main ORF using mRNA with 5′UTR of PKMζ. As a control we used mRNA with the same firefly luciferase coding sequence and short (86 b.) uORF-free 5′UTR of actin (**Figure 3A**). This mRNA had no hairpins and efficiently assembled the translation initiation complex on the ATG codon of the main frame. Therefore, we showed that the formation of the initiation complex on the start codon of the main frame in the presence of 5′UTR of PKMζ is strongly suppressed.

It is known that the eukaryotic initiation factor eIF4B amplifies helicase-like activity of eIF4A and it is necessary for translation of mRNAs with a complex secondary structure of 5′UTRs (Andreou and Klostermeier, 2013). To obtain additional data about the effect of the structure of 5′UTR on translation of PKMζ, we incubated eukaryotic initiation factors eIF4A and eIF4B in the RRL cell-free translation system with the wt and Δ1–7 mRNAs (**Figures 2C,D**). These factors demonstrated a moderate stimulatory effect on the efficiency of translation on both mRNAs. It also confirms the hypothesis that the structure of 5′UTR of PKMζ does not contribute significantly to its translation (see above).”

The truncated eIF4G (ΔeIF4G, p50) consists of the central third of eIF4G (aa 486–935) which interacts with both eIF3 and eIF4A and lacks PABP and eIF4E binding sites (De Gregorio et al., 1998). An addition of ΔeIF4G stimulates translation of 5′UTR of PKMζ (**Figure 2E**). On the contrary, this protein suppresses the translation of Δ1–7 mRNA. This is consistent with the earlier studies where p50 exerted (**Figure 2F**) a dominant negative effect on the translation of capped mRNAs and stimulated the translation of uncapped mRNAs (De Gregorio et al., 1998). Moreover, p50 strongly enhanced re-initiation in the eIF4G-depleted lysates (Pöyry et al., 2004).

Our results suggest that uORFs play the main role in the suppression of translation of PKMζ mRNA. To confirm our suggestion, we performed a toe-printing analysis of two mutant 5′UTRs of PKMζ, with AUG substituted by UAG in the first or second uORF (**Figure 3B**). On the wt 5′UTR of PKMζ, the 48S initiation complex assembles mainly on the first uORF, and we didn’t observe any ribosomal complex on AUG codon of the main ORF (**Figure 3A**). If the AUG codon of the first uORF was mutated (Δ1), 48S complexes were not registered in this position, but some complexes were shown to be located on the second uORF. On the other side, if the AUG codon of the second uORF was mutated (Δ2), initiation complexes were detected only on the first uORF (**Figure 3B**). Thus, we showed that uORFs within 5′UTR of PKMζ are indeed able to assemble the translation initiation complexes, and these uORFs may affect the translation from the main coding frame.

### uORFs in 5′UTR of PKMζ Inhibit Translation in Living Neurons

**FIGURE 4:**
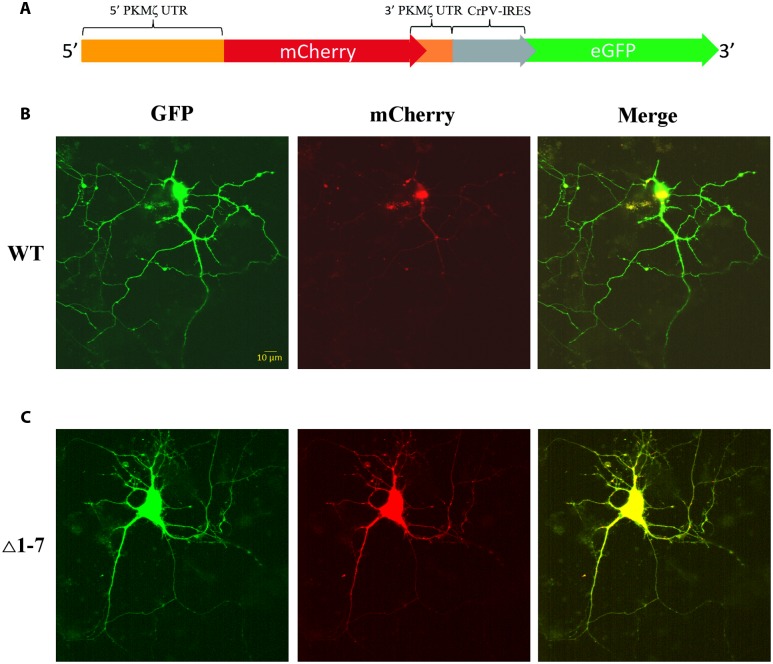
**Expression of mRNAs under control of regulatory elements of PKMζ 5′UTR in the primary neuron culture from rat hippocampus.** **(A)** Structure of genetic constructions used for expression of mRNA in neurons. Wild-type or mutated leaders of PKMζ are followed by mCherry gene under CMV promoter and GFP gene under control of CrPV IRES element. **(B)** Expression of mRNA with wt 5′UTR of PKMζ in the primary neuron culture from rat hippocampus. **(C)** Expression of mRNA with Δ1–7 5′UTR of PKMζ in the primary neuron culture from rat hippocampus. Expression of eGFP serves as a control, and reflects a possible level of protein synthesis without repression.

To investigate the PKMζ translation control in living cells we used a bicistronic system of two reporter fluorescent proteins that were inserted to the same plasmid (**Figure 4A**). The translation of the first protein, mCherry, was controlled by 5′UTR (wild-type or mutant) and 3′UTR of PKMζ. The second encoded protein, GFP, was controlled by the CPV-IRES-element that allows the 40S and 60S subunits to form 80S initiation complex without translation initiation factors and start translation immediately (**Figures 4B,C**) (Pestova et al., 2004). Therefore, the GFP translation was constitutive, and GFP fluorescence can be used for signal normalization.

**FIGURE 5:**
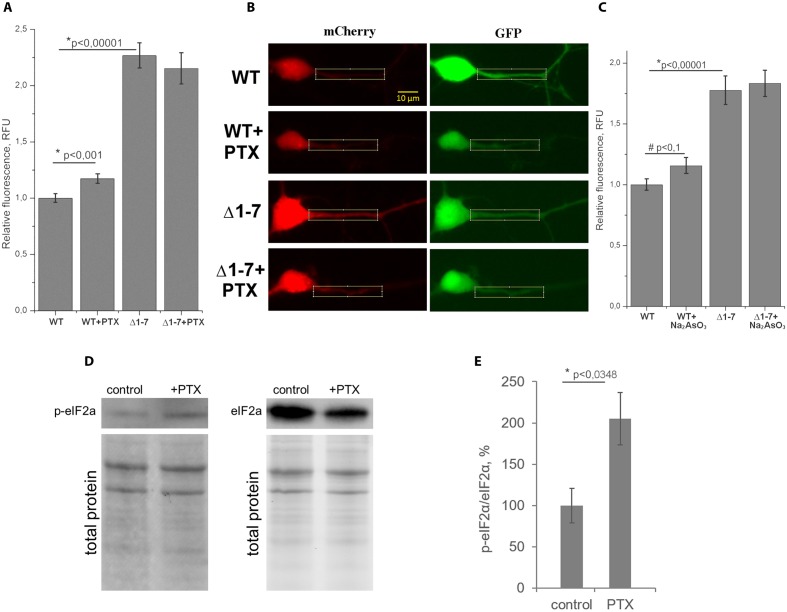
**Quantitative analysis of protein synthesis in primary neuron culture.** **(A)** Normalized ratios of mCherry/GFP fluorescence levels in cell cultures transfected by plasmids with wt or Δ1–7 5′UTR of PKMζ in the presence/absence of picrotoxin (PTX). **(B)** Examples of dendrites used for counting. **(C)** Normalized ratios of mCherry/GFP fluorescence levels in cell cultures transfected by plasmids with wt or Δ1–7 5′UTR of PKMζ in the presence/absence of Na_2_AsO_3_. **(D)** Western blot analyzes of the primary culture of rat neurons treated/untreated by PTX. Antibodies raised against eIF2α and eIF2α^P^ were used for detection of non-phosphorylated and phosphorylated forms of eIF2α, respectively. **(E)** Quantitative analysis of the western blots. eIF2a/eIF2a^P^ ratios normalized to total protein (per line). The error bars represent the standard error of mean, (*n* = 3).

On **Figure 5A** the normalized mCherry/GFP fluorescence level ratios in cell cultures transfected by plasmids with or without uORFs in PKMζ 5′UTR are shown. We found that the patterns of fluorescence were different in the cells transfected by plasmids containing wild-type or mutant 5′UTR of PKMζ (**Figures 4B,C**). In the case of the wild-type 5′UTR, synthesis of mCherry in dendrites was suppressed. On the contrary, the basic level of mCherry translation was high in the culture transfected by mutant Δ1-7 5′UTR of PKMζ. Using quantitative comparison (**Figures 5A,B**), we determined a twofold difference between fluorescence in the proximal parts of dendrites containing wt and mutant 5′UTRs of PKMζ (*p* < 0,00001, *N* = 80–86 neurons from three independent experiments). The changes in translation of mCherry caused by neuronal activation (we used picrotoxin to activate the cells) were also different in these two cultures. Namely, after activation the mCherry signal increased in wild-type PKMζ 5′UTR-containing neurons (*p* < 0,001, *N* = 58 neurons from three PTX-treated cultures), but didn’t change significantly in the cells that were transfected by plasmid carrying Δ1–7 5′UTR of PKMζ (*p* > 0,05; *N* = 42 from three independent cultures). Since mCherry fluorescence level correlates to the mCherry protein accumulation, we proved that uORFs are crucial for PKMζ translation. We also confirmed that translation of mRNA with wild-type 5′UTR of PKMζ increases after synaptic activation.

We suppose that the demonstrated increase of translation of mRNA with PKMζ regulatory elements in response to synaptic activation was caused by the eIF2α phosphorylation, which also occurs during cellular stress (Harding et al., 2000). To verify this hypothesis, we induced mild cellular stress by sodium arsenite in the cell cultures transfected by plasmids with or without uORFs in PKMζ 5′UTR (**Figure 5C**). Earlier it was shown that sodium arsenite induced stress is characterized by eIF2α phosphorylation and global translation arrest (McEwen et al., 2005; Tsai et al., 2009; Andreev et al., 2015). Our experiments showed that in the proximal parts dendrites of neurons containing the wild-type 5′UTR of PKMζ, the arsenite-induced stress causes a tendency for the relative fluorescence to increase (*p* < 0,1; *N* = 36–40 neurons in control and arsenite-treated group). In neurons containing the mutant 5′UTR of PKMζ, arsenite didn’t cause any significant changes in relative fluorescence (*p* > 0,05; *N* = 30–63 neurons). Therefore, we have concluded that translation of PKMζ mRNA in neurons may be associated with the eIF2α phosphorylation.

To further confirm our suggestion, we needed to check the level of eIF2α phosphorylation after activation of cells with picrotoxin. For this purpose, we determined the levels of the phosphorylated eIF2α (p-eIF2α) and non-phosphorylated eIF2α by immunoblotting in the same primary cell culture before and after picrotoxin activation. We found that the p-eIF2α/eIF2α concentration ratio is increased in the cells exposed to picrotoxin compared to a control culture that wasn’t activated (**Figures 5D,E**). Therefore, we have shown phosphorylation of eIF2α after the picrotoxin-induced activation of neurons, which confirms the supposed p-eIF2-dependent mechanism of activation of PKMζ translation.

## Discussion

There are various factors affecting local translation in a non-specific manner, in particular, ion concentrations. Our experiments performed *in vitro* showed that physiological concentrations of K^+^ and Mg^2+^ are suboptimal for PKMζ translation. Therefore, the maintenance of the normal cation concentrations in the cell may present another means for downregulation of the PKMζ synthesis (**Figures 2A,B**). The described influence of cation concentrations on the translation of PKMζ 5′UTR-carrying mRNA may be explained by the ability of cations to stabilize the mRNA secondary structure. Cations interact with the negatively charged ribose-phosphate backbone and prevent electrostatic repulsion between the mRNA chain fragments. It allows mRNA to form hairpin structures, which prevent binding and movement of the 48S initiation complex, hampering start codon recognition.

Some authors believe that the length of 5′UTR is an important factor in translation regulation of different neuron-specific proteins, PKMζ included (Hernandez et al., 2003; Eom et al., 2014). Seven uORFs in the PKMζ 5′UTR (**Figure 1A**) could be involved in the regulation of translation of this particular kinase (Chua et al., 2012; Di Prisco et al., 2014; Studtmann et al., 2014). We confirmed the role of these uORFs in the translational control of PKMζ. Using *in vitro* tests with reporter mRNAs carrying different numbers of uORFs in the 5′UTR we revealed that the main frame translation depends on the presence of uORFs (**Figure 1B**). Mutations in uORFs did not change the secondary structure of the leader sequence, but increased the translation rate. Thus, the structure of the 5′UTR is not critical for the translational control of PKMζ. Additionally, eukaryotic initiation factors eIF4A and eIF4B, known to improve initiation of translation on the structured mRNA, didn’t significantly facilitate translation on PKMζ 5′UTR (**Figures 2C,D**). Recently, it was supposed that eIF4B regulates the synthesis of PKMζ in neurons (Eom et al., 2014). Phosphorylation of S406 eIF4B increases its affinity to non-coding brain-specific BC1 RNA in rodents (or its analog BC200 RNA in primates). BC1 competes with 18S RNA for the translation initiation factors and thus ensures low translation levels in neurons (Lin et al., 2008). Therefore, eIF4B increases the general level of translation in neurons, but as we showed, it doesn’t directly influence the translation of PKMζ mRNA (**Figures 2C,D**).

However, addition of the truncated form of eIF4G into the cell-free system (**Figure 2E**) enhanced the efficiency of translation of mRNA with PKMζ 5′UTR. It was shown earlier that this form of eIF4G increases the probability of re-initiation events in cell-free systems on uORFs (Pöyry et al., 2004). Indeed, ΔeIF4G contains an eIF3 binding site and lacks the eIF4E binding region. Therefore, ΔeIF4G is able to interact with the 40S ribosomal subunit and to bind with mRNA via eIF3, but can’t be involved in the cap-dependent initiation of translation. This means that this protein can participate in re-initiation on uORFs and compete with the full-length eIF4G for the eIFs and 40S during the cap-dependent translation. Activation of translation on the PKMζ 5′UTR by ΔeIF4G (**Figure 2E**) confirms the suggestion on the crucial role of uORFs in translational control of the PKMζ synthesis.

The toe-printing analysis of the assembly of ribosomal complexes on the mRNA containing luciferase coding sequence and PKMζ 5′UTR in RRL showed the presence of a 48S complex on the first uORF (**Figure 3B**) and absence of the initiation complex on the main ORF (**Figure 3A**). On mRNA carrying PKMζ 5′UTR with UAG instead of AUG in the first uORF, the initiation complex was not registered in this position, but there was a very weak peak of the 48S complex on the second uORF. These results demonstrate that uORFs of PKMζ leader are indeed able to draw initiation complex components and may regulate the translation of mRNA.

We showed that uORFs are important for the regulation of the basic translation level of PKMζ in the cell (**Figure 4**). We also confirmed that the expression of PKMζ in a neuron may increase normally due to synaptic activation, and that uORFs are necessary for such a shift. GABA_A_R blockade by picrotoxin causes a lack of inhibiting signals from interneurons resulting in neuronal excitation. Such excitation is caused by excitatory postsynaptic potentials, similar to what happens normally in the living brain. When other models of cell activation in neuronal culture are used, such as KCl or glutamate/glycine application, excitation is caused by depolarization of a whole neuron. Therefore, we decided that the picrotoxin model is the most appropriate for our study in which local postsynaptic changes are of special interest. We have shown that picrotoxin-induced neuronal excitation results in an increase in translation of the reporter protein controlled by PKMζ 5′UTR, just as we expected (**Figure 5**).

Here we showed that uORFs in PKMζ 5′UTR repress translation of the reported protein in living cells (**Figures 4B,C**). A wide range of genes is known for which uORF-dependent suppression of translation occurs normally and this block is removed in cellular stress conditions. The key protein of this translational control is the initiation factor eIF2 (Costa-Mattioli et al., 2005; Bellato and Hajj, 2016). Phosphorylation of this protein switches between normal and uORF-dependent translation. There are at least two possible explanations for this fact, and other potential mechanisms may still be discussed.

It was shown that eIF2α phosphorylation is necessary for regulation of translation of proteins associated with LTD (long-term synaptic depression). It was supposed that during LTD, eIF2α phosphorylation causes overall translation inhibition and corresponding activation of expression of some proteins coded by the uORF-containing mRNAs (Di Prisco et al., 2014). For the uORF-containing mRNAs, initiation is hindered and represents a rate-limiting step in the whole translation process. But in this case, once initiation has happened, there are not so many impediments for elongation or termination steps. Therefore, in such conditions when the initiation is hardly likely for all mRNAs, the uORF-containing mRNAs get a relative advantage in translation because of their comparatively easy elongation and termination.

Another mechanism of the LTD-associated translation switch has been proposed (Graber et al., 2013). It was demonstrated that the “stalled” initiation ribosome complexes containing mRNA of the LTD-associated proteins are present in dendrites. The translation from these complexes started after mGluR activation and could be prevented by antibiotics that affect elongation, but not the initiation step. It was thought that the initiation stage of such mRNAs happens in the soma, and they are ready for elongation without further delay and may not be stopped by the initiation blockade that happens if eIF2α is phosphorylated.

In our study, we propose that local changes in PKMζ translation in dendrites caused by synaptic activation are regulated by mechanisms that are similar to the transient response to cell stress. We have shown that there was a tendency for an increase in translation of the mRNA carrying PKMζ regulatory elements in the case of arsenite-induced stress (**Figure 5C**). It is known that (Tsai et al., 2009; Andreev et al., 2015) arsenite disrupts redox equilibrium in the cell and causes EPR (endoplasmic reticulum) malfunction. These negative effects lead to eIF2α phosphorylation and to the formation of stress granules. Stress granules are ribonucleoprotein complexes that contain the mRNAs which translation is arrested (Tsai et al., 2009). Both eIF2α phosphorylation and stress granule formation decrease the basic translation level but favor the translation from mRNAs containing the uORFs in 5′UTR (Andreev et al., 2015). This explains the increase in the reporter protein translation that we have measured after stress induction (**Figure 5**).

We suppose, that with the condition of low arsenite concentration that we used (**Figure 5C**), the stress was mild and the eIF2α phosphorylation was transient, similar to the EPR stress induced by thapsigargin, (Jakobson et al., 2013). Such transient eIF2α phosphorylation possibly occurs during the learning processes (Cho et al., 2015). It was demonstrated in mice that in the first 5 min after learning there was a decrease of the overall translation that continued for approximately 30 min. Most proteins, for which synthesis was repressed at this period, were encoded by mRNAs that require cap binding and initiation complex assembly for their translation. In the animals with knockout of the eIF2α kinase genes, or when the eIF2α kinase inhibitors are used, this 30-min decrease in translation is apparently absent. Temporary eIF2α phosphorylation is believed to be very important for the proper formation of the synaptic network in learning, because its blockade increases general the excitability of neurons and disrupts the “fine tuning” of learning processes (Zhu et al., 2011).

Bioinformatics analysis showed that in the nervous system there are more genes expressed, whose mRNA contains uORFs, in comparison to the full transcriptome of mice (**Supplementary Figure S1A**). Interestingly, learning (training) of animals leads to an increase in translation 5 min after stimuli, according to the ribo-seq of mRNA with uORFs (**Supplementary Figure S1B**) (Cho et al., 2015). This indicates, that uORF can regulate translation during learning not only in the case of mRNA of PKMζ.

When the overall translation is repressed, mRNAs with uORFs in 5′UTR gain a relative advantage in translation. The phosphorylated eIF2α accumulates both during neuronal activation (Costa-Mattioli et al., 2007, 2009) and in stress (Harding et al., 2000). Our experimental data confirmed that translation of the mRNA with PKMζ 5′UTR activates with the increase of the eIF2α^P^ concentration, both in the presence of picrotoxin and sodium arsenite (**Figures 5A,C**). Moreover, we showed that the uORFs are crucial for such an increase, because point mutations affecting the uORFs attenuated this effect. Therefore, our results suggest that one of the mechanisms of translational control of PKMζ is by phosphorylation of the eIF2α.

## Author Contributions

Study conception and design by PK, PB, and EA. NB, DS, EC, AK, and TM conducted the experiments. PK, PB, and EA wrote the article.

## Conflict of Interest Statement

The authors declare that the research was conducted in the absence of any commercial or financial relationships that could be construed as a potential conflict of interest.

## Supplementary Material

**FIGURE S1 | Computational analysis of uORF distribution in neuronal transcriptome.** **(A,B)** Percentage of mRNAs with 0–2 and 3–10 uORFs in **(A)** non-neuronal and neuronal transcriptomes (χ_2_*p*-value < 0.0001) and **(B)** hippocampal 5 min after learning Ribo-seq data set and neuronal mouse transcriptome (χ_2_ *p*-value = 0.0287).
