# Supplementary figures and images for: Upstream Open Reading Frames Located in the Leader of Protein Kinase Mζ mRNA Regulate Its Translation
Source: Front Mol Neurosci. 2016 Oct 13;9:103. doi: 10.3389/fnmol.2016.00103 (PMC5061749; doi:10.3389/fnmol.2016.00103)

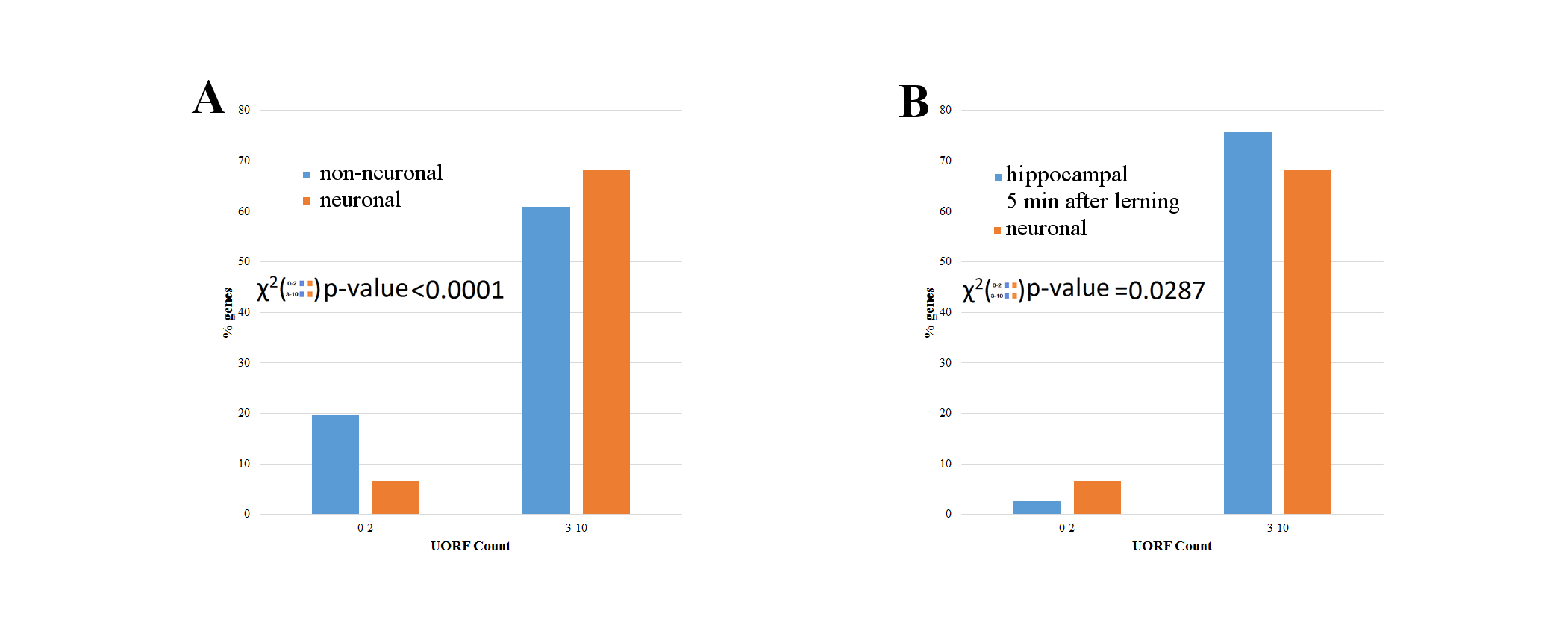

Supplement: Supplementary file 1 [file Image_1.TIF]
